# The Dynamics of Inflammatory Markers in Patients with Suspected Acute Appendicitis

**DOI:** 10.3390/medicina57121384

**Published:** 2021-12-20

**Authors:** Ąžuolas Algimantas Kaminskas, Raminta Lukšaitė-Lukštė, Eugenijus Jasiūnas, Artūras Samuilis, Vytautas Augustinavičius, Marius Kryžauskas, Kęstutis Strupas, Tomas Poškus

**Affiliations:** 1 Faculty of Medicine, Vilnius University, LT-03101 Vilnius, Lithuania; azuolas.kaminskas@gmail.com; 2 Department of Radiology, Nuclear Medicine and Medical Physics, Institute of Biomedical Sciences, Faculty of Medicine, Vilnius University, LT-08661 Vilnius, Lithuania; arturas.samuilis@santa.lt (A.S.); Vytautas.Augustinavicius@santa.lt (V.A.); 3 Center of Informatics and Development, Vilnius University Hospital Santaros Klinikos, LT-08661 Vilnius, Lithuania; Eugenijus.Jasiunas@santa.lt; 4 Clinic of Gastroenterology, Nephro-Urology, and Surgery, Institute of Clinical Medicine, Faculty of Medicine, Vilnius University, LT-08661 Vilnius, Lithuania; marius.kryzauskas@santa.lt (M.K.); kestutis.strupas@santa.lt (K.S.); Tomas.Poskus@santa.lt (T.P.)

**Keywords:** acute appendicitis, laboratory tests, inflammatory markers, complicated acute appendicitis, non-complicated acute appendicitis

## Abstract

*Background*: Laboratory tests of inflammatory mediators are routinely used in the diagnosis of acute appendicitis (AA). The aim of this study was to evaluate the differences of dynamics of inflammatory markers of the blood in patients with suspected acute appendicitis between complicated AA (CAA), non-complicated AA (NAA), and when AA was excluded (No-AA). *Methods*: This was a retrospective analysis of prospectively collected data of patients presented to the Emergency Department (ER) of a tertiary hospital center during a three-year period. All patients suspected of acute appendicitis were prospectively registered from 1 January 2016 to 31 December 2018. The dynamics of inflammatory markers of the blood between different types of AA (No-AA, NAA or CAA) during different periods of time are presented. *Results*: A total of 453 patients were included in the study, with 297 patients in the No-AA group, 99 in the NAA group, and 57 in the CAA group. White blood cell (WBC) count in the No-AA decreased with time, with a statistically significant difference between the <8 h and 25–72 h group. The neutrophils (NEU) percentage decreased in the No-AA group and was statistically significantly different between the <8 h and 25–72 h and <8 h and >72 h groups. C-reactive protein (CRP) increased significantly in the No-AA group throughout all time intervals, and from the first 24 h to the 25–72 h in the NAA and CAA groups. There was a statistically significant difference between the WBC count between No-AA, NAA, and No-AA and CAA groups during the first 24 and 24–48 h. There was a statistically significant difference between NEU percentage and LYMP percentage and in the NEU/LYMP ratio between No-AA and CAA groups through all time periods. CRP was significantly higher in the first 24 h in the CAA than in the No-AA group, and in the 24–48 h in the CAA group than in the No-AA and NAA groups. The linear logistic regression model, involving inflammatory mediators and clinical characteristics, showed mediocre diagnostic accuracy for diagnosing AA with an AUC of 0.737 (0.671–0.802). *Conclusions*: Increasing concentrations of inflammatory markers are more characteristic in CAA patients than in No-AA during the first 48 h after onset of the disease. A combination of laboratory tests with clinical signs and symptoms has a mediocre diagnostic accuracy in suspecting AA.

## 1. Introduction

Acute appendicitis (AA) is one of the most common causes of acute abdomen in adults, with 17.7 million cases worldwide in 2019 and an incidence of 228 cases per 100,000 population [1,2]. Early diagnosis of AA, and proper identification of the type of AA, are crucial to achieve optimal treatment results. Strategies for complicated (CAA) and non-complicated (NAA) AA treatments differ [3]. If CAA is not diagnosed and treated early, serious complications can develop; on the other hand, NAA can be treated conservatively [3,4,5]. Clinical and laboratory scores can be used to rule out the diagnosis of appendicitis [3,6]. However, AA remains diagnostically challenging, as the negative appendectomy (NA) incidence can reach up to 10–30% [7,8,9,10].

The clinical diagnosis is confirmed and the specific type of AA (CAA or NAA) is diagnosed with imaging studies, such as transabdominal ultrasound (TUS) and computed tomography (CT) scan, or MRI scan in pregnant women [11]. Their use has been associated with reduced NA rates [12,13]. Nonetheless, TUS is a subjective diagnostic tool with its middling sensitivity and specificity (about 77% and 60%, respectively), as well as the limited ability to visualize the appendix (normal appendix is detected in 71% of cases) [14,15]. CT has high sensitivity and specificity (about 90–95% and 94%, respectively) in visualizing AA, however, it uses ionizing radiation and is associated with an increased risk of future oncological diseases, especially in young patients [16,17,18]. Conditional use of CT scanning after US could be a possible solution for this problem [19].

Another possible tool that could be used to suspect, diagnose, or even differentiate AA types is laboratory tests. Some studies have shown a possible correlation between an elevated concentration of inflammatory markers of the blood and the diagnosis of AA [20]. On the other hand, the absence of inflammatory changes in the blood cannot exclude the diagnosis of AA [21,22]. Furthermore, the literature suggests that inflammatory changes in the blood could be used to diagnose a specific type of AA (CAA or NAA) [23]. However, there are limited data in the literature on the use of laboratory tests in the diagnosis of AA compared to the number of studies performed on imaging examinations. The aim of this study was to evaluate the differences in the dynamics of inflammatory markers of the blood in patients with suspected acute appendicitis between complicated AA (CAA), non-complicated AA (NAA), and when AA was excluded (No-AA).

## 2. Materials and Methods

This is a pre-planned analysis of inflammatory markers’ data, which were obtained from a cohort of patients from our previous research [19]. In short, all patients suspected of acute appendicitis were prospectively registered from 1 January 2016 to 31 December 2018. All patients over 18 years of age who were admitted to the emergency department because of symptoms suggestive of AA and consulted by a general surgeon were enrolled into the database. Only pregnant women were excluded from the database. All patients from the database underwent transabdominal ultrasound (TUS) and CT scan later if TUS was inconclusive and clinical suspicion of AA was still present. The TUS criteria for probable radiological diagnosis of AA were a diameter of appendix at ~7 mm (or less), wall thickness of the appendix at ~2 mm (or less), compressible/partially compressible appendix with or without secondary findings of free fluid in right iliac fossa, lymphadenopathy, and infiltration of surrounding tissue. The CT scan criteria for radiological diagnosis of AA were diameter of appendix ≥7 mm and wall thickness of the appendix ≥2 mm, with possible secondary signs of free fluid in right iliac fossa, lymphadenopathy, and fat stranding. Patients who underwent both CT and US to rule out AA were included in the study. The following information was collected and analysed in this study: white blood cell count (WBC), percentage of neutrophils (NEU), percentage of lymphocytes (LYMP), percentage of monocytes (MON), percentage of eosinophils (EOS), percentage of basophils (BAS), and concentration of C-reactive protein (CRP). All patients included in this study were divided into three groups based on their final diagnosis: complicated acute appendicitis (CAA) group, non-complicated acute appendicitis (NAA) group, and no appendicitis (No-AA) group. The final diagnosis of each patient was determined by a panel of experts based on histopathology, imaging, surgical findings, and clinical information.

The histological criteria for diagnosing AA were the following:

- Catarrhal appendicitis—neutrophils within mucosa and mucosal ulceration, with or without intraluminal neutrophils;
- Secondary changes/periappendicitis—inflammation of serosa and subserosa, infiltration extends no further than outer muscularis propria;
- Phlegmonous appendicitis—neutrophilic infiltration of mucosa, submucosa, and muscularis propria; transmural inflammation; extensive ulceration and intramural abscesses; vascular thrombosis;
- Gangrenous appendicitis—transmural inflammation with areas of necrosis, extensive mucosal ulceration.

The surgical findings for suspecting AA were the following:

- Catarrhal appendicitis—no visible changes;
- Secondary changes/periappendicitis—may appear normal or serosa may be dull, congested, and show exudate;
- Phlegmonous appendicitis—dilated or increased diameter appendix; dull serosa; dilatation and congestion of surface vessels; fibrinopurulent serosal exudate;
- Gangrenous appendicitis—appendiceal wall friable; purple, green, or black.

We did not define specific clinical criteria for the diagnosis of acute appendicitis (i.e., we suspected AA based on classical clinical signs and symptoms).

Only those patients who showed signs of AA in the CT scan were operated on, with a few exceptional cases when patients underwent surgery without visible signs of AA in CT scan. In addition, some CAA patients also did not have surgery—they underwent percutaneous drainage and conservative treatment.

The dynamics of inflammatory markers of the blood were evaluated at different time intervals: I (less than 8 h from the onset of the disease), II (8–16 h after the onset of the disease), III (17–24 h), IV (25–72 h), and V (more than 72 h after the beginning of the illness).

### Statistical Analysis

R statistical software package Version 4.0.5 (© The R Foundation for Statistical Computing, Vienna, Austria), Rstudio Version 1.2.5042 (© 2009–2021 RStudio, Inc., Boston, MA, USA), IBM SPSS Statistics V.23, and G*Power V. 3.1.9.4 Universität Düsseldorf, Germany were used for data analysis. Interval and ratio variables were described as medians, first (Q1) and third (Q3) quartiles, and interquartile range (IQR 75%). Shapiro–Wilk and Anderson–Darling tests were used to check the normality of the data. Nominal variables were described by their repeatability and percentage of the corresponding subgroup of the sample. Nonparametric Kruskal–Wallis H test was used to evaluate the dependence of several independent interval or rank variable samples. Epsilon squared (ε^2^) was used to measure the effect size (when ε^2^ is considered: 0.00–0.01—negligible effect size, 0.01–0.04—weak effect size, 0.04–0.16—moderate effect size, 0.16–0.36—relatively strong effect size, 0.36–0.64—strong effect size, 0.64–1.00—very strong effect size; according to Rea and Parker (1992)). Dunn’s test was used to determine statistically significant relationships between pairs of variables. To determine the dependence and its strength between nominal and categorical variables, the Chi-squared test and Cramér’s V (φ_c_) (when data are described in n × k type tables) effect sizes with their associated *p* values were used. According to Cohen (1988) (Table 1):

The Youden criterion was used for determining the optimal cut points for values of the laboratory indicators. A prognostic model was developed by using multivariate logistic regression. The pseudo-coefficient of determination was calculated by using more liberal Cragg–Uhler and more rigorous McFadden methods (McFadden’s pseudo R^2^ ranging from 0.2 to 0.4 indicates a very good model fit). In addition to pseudo-coefficient of determination, sensitivity, specificity, positive and negative predictive values, and area under the curve (AUC or AUROC) were provided for model accuracy assessment. The classification ability of the model was considered good when AUROC was >0.7 but <0.8, and AUROC > 0.8 was considered as excellent classification ability of the model. To test the hypotheses, we selected the significance of statistical tests α = 0.05 (*p* value <0.05) and the power of statistical tests 1 − ß = 0.95.

## 3. Results

Out of the 1855 patients in the database, 453 patients were included in the study, with 297 patients in the No-AA group, 99 in the NAA group, and 57 in the CAA group. Their clinical and demographic characteristics are presented in Table 2.

### 3.1. Dynamics of Inflammatory Markers within No-AA, NAA, and CAA Groups

**Figure 1:**
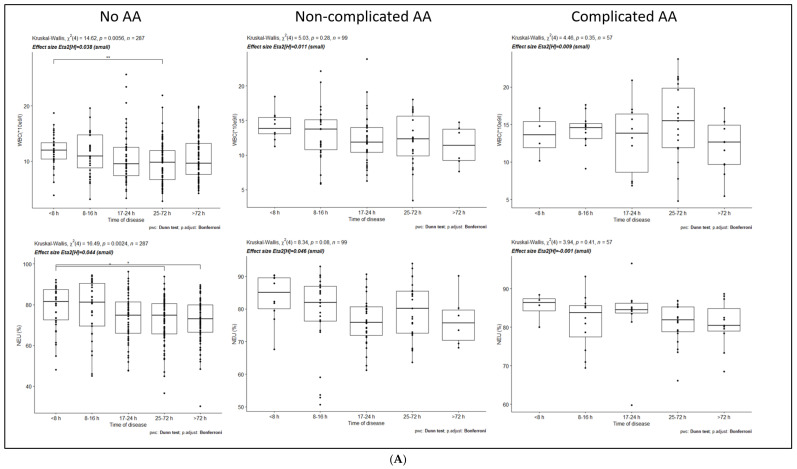

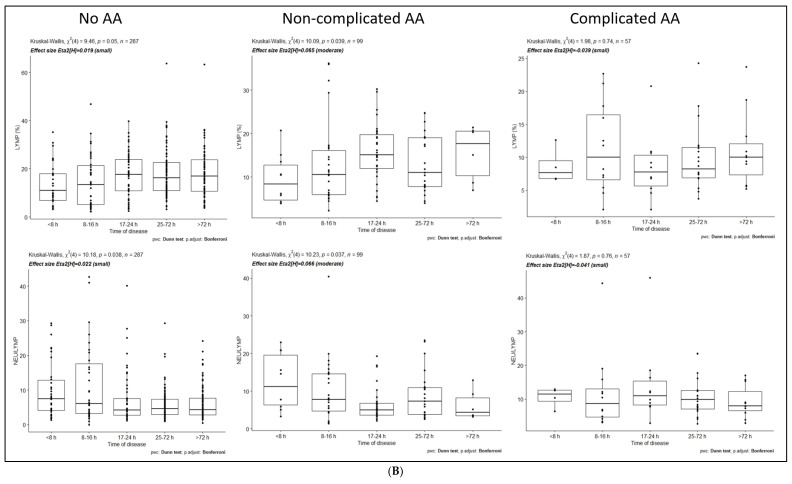

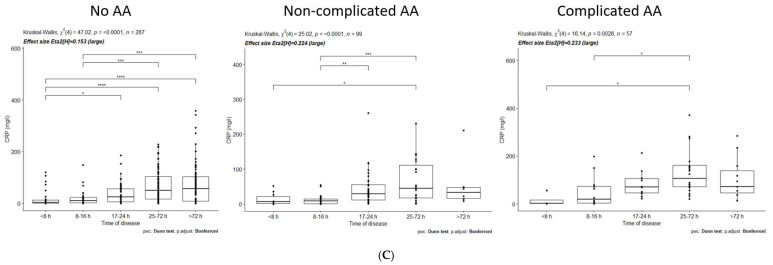
(**A**). No-AA—no acute appendicitis group, NAA—non-complicated acute appendicitis group, CAA—complicated acute appendicitis group, WBC—white blood cell, NEU—neutrophils. (**B**). No-AA—no acute appendicitis group, NAA—non-complicated acute appendicitis group, CAA—complicated acute appendicitis group, LYMP—lymphocytes, NEU/LYMP—neutrophil to lymphocyte ratio. (**C**). No-AA—no acute appendicitis group, NAA—non-complicated acute appendicitis group, CAA—complicated acute appendicitis group, CRP—C-reactive protein. *, **, *** and ****—indicates statistically significant differences between groups.

Figure 1A–C show the overall dynamics of laboratory tests in three study groups at five different time intervals.

Statistically significant differences between different time periods within the groups and their values are presented (Table 3).

The WBC count in the No-AA group is decreasing with time, with a statistically significant difference between <8 h and 25–72 h group (*p* value <0.05). The WBC count is not statistically significantly different in other groups and time periods. NEU percentage decreases in the No-AA group and is statistically significantly different between the <8 h and 25–72 h and <8 h and >72 h groups. No significant differences are observed between LYMP percentage and NEU/LYMP ratios between the groups. CRP increases significantly in the No-AA group throughout all time intervals, and from the first 24 h to the 25–72 h in the NAA and CAA groups.

### 3.2. Comparison of Inflammatory Markers Dynamics between No-AA, NAA, and CAA Groups

**Figure 2:**
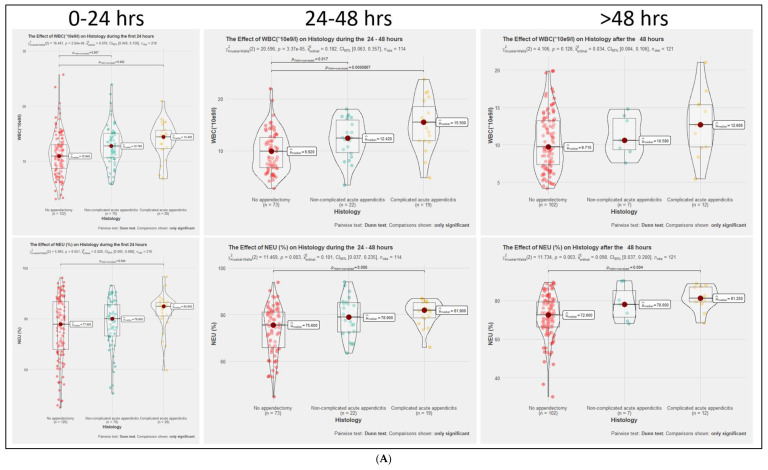

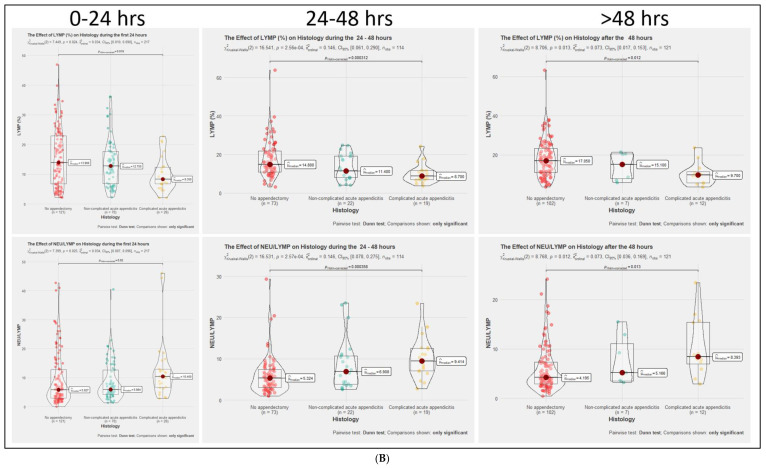

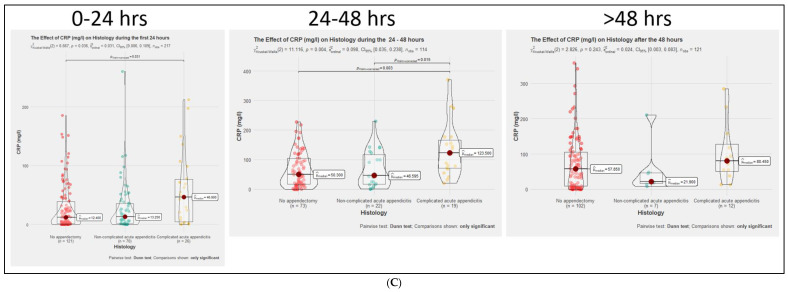
(**A**). No-AA—no acute appendicitis group, NAA—non-complicated acute appendicitis group, CAA—complicated acute appendicitis group, WBC—white blood cell, NEU—neutrophils. (**B**). No-AA—no acute appendicitis group, NAA—non-complicated acute appendicitis group, CAA—complicated acute appendicitis group, LYMP—lymphocytes, NEU/LYMP—neutrophil to lymphocyte ratio. (**C**). No-AA—no acute appendicitis group, NAA—non-complicated acute appendicitis group, CAA—complicated acute appendicitis group, CRP—C-reactive protein.

Figure 2A–C present the comparison of the groups by levels of inflammatory markers during different time periods.

There is a statistically significant difference between the WBC count between No-AA, NAA, and No-AA and CAA groups during the first 24 and 24–48 h. There is a statistically significant difference between NEU percentage and LYMP percentage and in the NEU/LYMP ratio between No-AA and CAA groups through all time periods. CRP is significantly higher in the first 24 h in the CAA than in the No-AA group, and in the 24–48 h in the CAA group than in the No-AA and NAA groups.

### 3.3. Linear Logistic Regression Model on Inflammatory Markers and the Diagnosis of AA

**Figure 3:**
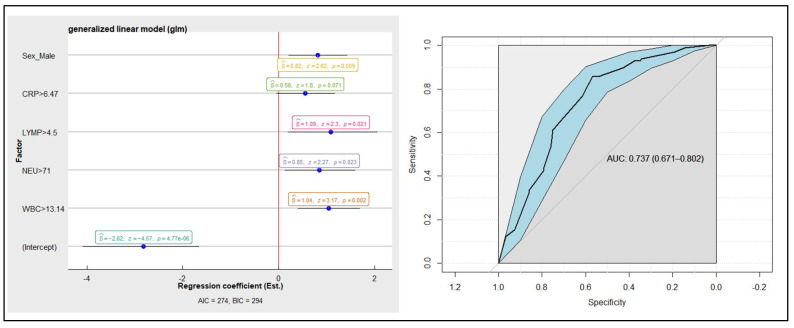
Linear logistic regression model with area under the curve (AUC) presented graphically.

A linear logistic regression model was created to differentiate between combined AA groups and the No-AA group (Figure 3). The AUC of the model for diagnosing acute appendicitis is 0.737 (0.671–0.802).

## 4. Discussion

We found that some inflammatory markers differ within groups of patients, with varied duration from the onset of symptoms. The biggest changes were seen with the increase in the CRP concentration in all groups and the decrease in the WBC count and the NEU in the NAA group.

We also found that WBC levels are higher in both NAA and CAA groups than in the No-AA group within the first 48 h. The NEU percentage and the NEU/LYMP ratio are higher in the CAA than in the No-AA group and, respectively, the LYMP percentage is higher in the No-AA group than in the CAA group, despite the duration of the disease. CRP is higher in the CAA than in the No-AA group within the first 24 h, and higher than in both the No-AA and NAA groups within 24–48 h.

The diagnostic model, involving only inflammatory mediators and clinical characteristics, can accurately diagnose AA in 73% of cases.

This is a prospective real-world cohort study, where all the patients with suspected AA were included and followed up to one month after their initial visit to confirm the diagnosis. However, the study has several drawbacks. All data were collected at the single tertiary center, so the results may differ from situations in other treatment facilities. All blood samples were taken and analysed according to the usual hospital procedures, in the absence of a specific study protocol defining how samples should be taken, transported, and analysed. Furthermore, in this study, we only analysed the dynamics of inflammatory markers between different patients in different groups, however, we did not analyse the changes in inflammatory markers in the same patients, as could be performed in patients under observation.

This study confirms the results of previous research, where the diagnostic accuracy of inflammatory mediators reaches 75% [7,24,25,26]. The addition of a conditional CT protocol in this group of patients resulted in a diagnostic accuracy of 96% and the overall use of CT of 29% for diagnosis [19]. This is very similar to other series, where the use of CT scans resulted in high diagnostic effectiveness [12,16,17,27,28,29,30].

Nevertheless, as suspicion of AA is common in young adults, the increased future cancer risk is an important consideration. There is a need for non-radioactive, repeatable diagnostic tools for AA, and inflammatory markers are one of them. One of the main areas where laboratory tests could be very useful is a triage of the patients and referral to imaging examinations. A prospective observational study was performed to evaluate the accuracy of a diagnostic panel of laboratory tests which consisted of WBC, CRP, and myeloid related protein 8/14 (MRP 8/14) [31]. It was found to have a sensitivity of 97.5% (95% CI, 91.3–99.3%), negative predictive value of 98.4% (95% CI, 94.4–99.6%), and a negative likelihood ratio of 0.07 (95% CI, 0.02–0.27). A systematic review with meta-analysis revealed that neutrophil-to-lymphocyte (NEU/LYMP) ratio could possibly be used for identification of AA and prediction of its severity (NAA or CAA) [23]. Binary logistic regression analysis showed that a NEU/LYMP ratio greater than 4.7 was an independent predictor of AA, with sensitivity of 88.89% (95% CI 70.8–97.6%), specificity of 90.91% (95% CI, 58.7–99.8%), and with a high accuracy (AUC) of 0.96 (95% CI 0.84–1.0, *p* < 0.0001). Additionally, NEU/LYMP ratio >8.8 was identified as independent predictor of CAA with sensitivity of 76.92% (95% CI, 46.2–95.0%) and specificity of 100% (95% CI, 75.3–100%) with AUC being 0.91 (95% CI 0.73–0.99, *p* < 0.0001). Another prospective observational study has shown that it is very unlikely that patients with normal concentrations of inflammatory markers will have AA (negative predictive value reached 95% when WBC count value was within normal range alone, and 100% when both WBC and CRP concentrations were normal) [32]. Normal inflammatory marker values are very unlikely in cases of AA, and laboratory tests may be appropriate to initially rule out the diagnosis of AA [25,33,34,35]. Therefore, when inflammatory markers’ values are within normal ranges, a watchful waiting could be chosen, which in turn could reduce the excessive use of CT. In our study, we did not estimate optimal cut-off values for inflammatory indicators which would be reliable to confirm the diagnosis of AA. Furthermore, we did not analyse the accuracy of inflammatory markers in rejecting the diagnosis of AA. However, we noticed that patients in the No-AA group had a significantly lower WBC count and CRP concentration compared to the CAA group during the first 24 and 24–48 h after onset of the disease, and these results are similar to the findings of other studies, mentioned earlier.

According to the 2020 update of the WSES Jerusalem guidelines, the antibiotic-first strategy can be considered safe and effective in selected patients with uncomplicated acute appendicitis [3]. Based on this statement, our previously mentioned observation could theoretically suggest that patients with symptoms similar to AA, but with low WBC and CRP levels, could possibly have NAA or no AA at all, and, in that case, they could be carefully selected and treated conservatively without the need for excessive use of CT.

Changes in the concentrations of individual inflammatory indicators are not sufficiently accurate in suspecting or excluding the diagnosis of AA [25]. However, when combined with each other or with other clinical examinations, the accuracy of laboratory tests in diagnosing AA increases [20,25,34]. Combinations of inflammatory marker values with each other or with clinical symptoms and signs can effectively contribute to ruling out the diagnosis of AA and triaging patients who are suspected to have AA [6,31]. Many clinical scores that can be used in the diagnosis of AA are reported in the literature. Alvarado, Appendicitis Inflammatory Response (AIR), Adult Appendicitis Score (AAS), Raja Isteri Pengiran Anak Saleha Appendicitis (RIPASA), and many other AA diagnostic scores combine certain clinical signs and symptoms with inflammatory markers [6,36,37,38]. These scores are used in clinical practice, and some of the most studied and most often included in the international guidelines for AA are Alvarado, AIR, and ASS scores [3]. The Alvarado score is suitable for rejecting the diagnosis of AA when the cut-off point is less than 5 (sensitivity of 99%) but not accurate enough to “rule-in“ the diagnosis of AA [6]. The AIR score has been shown to be the best clinical predictor (92% sensitivity and 63% specificity) of AA among other scores [39]. The 2020 update of the WSES Jerusalem guidelines recommends against using the Alvarado score to positively confirm the clinical suspicion of AA in adults, and to use the AIR score and AAS score as clinical predictors of AA [3]. Moreover, the addition of laboratory tests’ results may increase the accuracy of imaging studies when AA is suspected—a retrospective cohort study demonstrated that the combination of ultrasound and WBC count with the polymorphonuclear leukocyte differential can considerably improve the predictive value of ultrasound in diagnosing AA in children (with positive predictive value reaching up to 96.8%, and negative predictive value reaching up to 100% in certain cases) [40]. Our linear logistic regression model, which involved only inflammatory mediators and clinical characteristics, showed moderate diagnostic accuracy, with an AUC of 0.737 (0.671–0.802). We did not analyse how changes in inflammatory indicators could affect the accuracy of ultrasound in diagnosing AA, but this could be a useful topic for future research. However, we noticed that WBC count, NEU, and CRP were significantly higher in the CAA group than in the No-AA group during the first 48 h from the onset of the disease. For future research, it could be useful to examine whether these three inflammatory markers combined have greater diagnostic accuracy in suspecting AA than each alone.

In addition to classical inflammatory markers (such as leukocytes and CRP) used in the evaluation of diagnostics of AA, the literature indicates other new and innovative inflammatory markers which could be potentially used in diagnosing AA. Possible utilization of bilirubin, ischemia-modified albumin, interleukin-6, serum amyloid A, matrix metalloproteinase, myeloperoxidase, calprotectin, serum fibrinogen, and other laboratory markers in the diagnostics of AA were described in the literature [25,41,42,43,44,45,46,47]. However, the application of a significant proportion of these laboratory tests in clinical practice is limited because smaller medical institutions (which are not tertiary centers) do not have the technological capacity to identify these indicators. In addition, a significant proportion of these inflammatory markers are characterized by low to moderate sensitivity and specificity as well as middling prognostic accuracy in suspecting AA [25,26]. Two meta-analyses showed the high diagnostic accuracy of procalcitonin in suspecting CAA. Procalcitonin’s positive likelihood ratio in diagnosing CAA reached 9.53 (sensitivity—62%, specificity—94%, AUC—0.94) in one study [48], and the diagnostic odds ratio of procalcitonin in diagnosing CAA reached 76.7 (2.1, 272.9) in other research (sensitivity—89%, specificity—90%, AUC—0.96) [49]. In our study, for the evaluation of laboratory tests’ dynamics, we used a classical panel of inflammatory markers (consisting of WBC count, NEU, LYMP, NEU/LYMP, and CRP). Based on the observations of these two meta-analyses, for future work it may be useful to include procalcitonin in the list of laboratory tests to be analysed.

This study demonstrated that increased concentrations of inflammatory markers are more characteristic in CAA patients than in No-AA patients during the first 48 h after onset of the disease. Furthermore, a combination of laboratory tests with clinical signs and symptoms has a mediocre diagnostic accuracy in suspecting AA.

## 5. Conclusions

To conclude, we found that elevated inflammatory markers are more likely to be present in cases of complicated acute appendicitis during first 48 h after onset of the disease than in other conditions when the appendix remains normal. Laboratory tests alone or in combination with other clinical examinations could be a useful diagnostic tool in suspecting acute appendicitis, however, more research is needed to elucidate the exact role of inflammatory markers in the diagnostics of acute appendicitis.

## Figures and Tables

**Table 1:** The guidelines according to Cohen (1988).

Df *	Negligible	Small	Medium	Large
1	0–0.1	0.1–0.3	0.3–0.5	0.5 or more
2	0–0.07	0.07–0.21	0.21–0.35	0.35 or more
3	0–0.06	0.06–0.17	0.17–0.29	0.29 or more
4	0–0.05	0.05–0.15	0.15–0.25	0.25 or more
5	0–0.05	0.05–0.13	0.13–0.22	0.22 or more

Df *—Degrees of freedom (number of variables—1).

**Table 2:** Demographic and clinical characteristics of patients according to three main time intervals from the onset of the disease.

Overall (*n* = 453)									
Type of Disease	No-AA (*n* = 297)			NAA (*n* = 99)			CAA (*n* = 57)		
Time of disease (hours)	≤24 h	25–48 h	>48 h	≤24 h	25–48 h	>48 h	≤24 h	25–48 h	>48 h
Age (years)	28 (18–91)	38 (19–85)	40 (18–89)	30 (18–72)	31 (22–76)	32 (21–39)	48 (19–83)	49 (21–78)	48 (23–77)
Laboratory tests:									
WBC (*10e9/L)	11 (3–26)	10 (3–22)	10 (4–20)	13 (6–24)	12 (3–18)	11 (8–15)	14 (7–21)	16 (5–24)	13 (5–21)
NEU (%)	78 (45–96)	76 (45–94)	73 (30–90)	80 (51–93)	79 (64–94)	78 (68–90)	85 (60–97)	82 (66–87)	81 (68–89)
LYMP (%)	14 (2–47)	15 (3–64)	17 (4–63)	13 (2–36)	11 (4–24)	15 (6–21)	8 (2–23)	9 (4–24)	10 (4–24)
NEU/LYMP	6 (0–43)	5 (1–29)	4 (0–24)	6 (1–40)	7 (3–24)	5 (3–16)	10 (3–46)	9 (3–23)	8 (3–23)
CRP (mg/L)	12 (0–185)	50 (0–228)	58 (0–358)	13 (0–260)	47 (0–230)	22 (9–210)	47 (1–212)	124 (20–371)	80 (13–284)

No-AA—no acute appendicitis group, NAA—non-complicated acute appendicitis group, CAA—complicated acute appendicitis group, WBC—white blood cell, NEU—neutrophils, LYMP—lymphocytes, NEU/LYMP—neutrophil to lymphocyte ratio, CRP—C-reactive protein.

**Table 3:** Statistically significant differences between different time periods within the groups and their values.

Group	Parameter	Time Period (1)	Time Period (2)	*p*-Value	Adjusted *p*-Value
**No-AA**	WBC (*10e9/L)	<8 h	25–72 h	0	0.01
	NEU (%)	<8 h	25–72 h	0	0.03
		<8 h	>72 h	0	0.02
	LYMP (%)	No statistically significant differences between time intervals			
	NEU/LYMP	No statistically significant differences between time intervals			
	CRP (mg/L)	<8 h	17–24 h	0	0.05
		<8 h	25–72 h	0	0
		<8 h	>72 h	0	0
		8–16 h	25–72 h	0	0
		8–16 h	>72 h	0	0
**NAA**	WBC (*10e9/L)	No statistically significant differences between time intervals			
	NEU (%)	No statistically significant differences between time intervals			
	LYMP (%)	No statistically significant differences between time intervals			
	NEU/LYMP	No statistically significant differences between time intervals			
	CRP (mg/L)	<8 h	25–72 h	0	0.04
		8–16 h	17–24 h	0	0
		8–16 h	25–72 h	0	0
**CAA**	WBC (*10e9/L)	No statistically significant differences between time intervals			
	NEU (%)	No statistically significant differences between time intervals			
	LYMP (%)	No statistically significant differences between time intervals			
	NEU/LYMP	No statistically significant differences between time intervals			
	CRP (mg/L)	<8 h	25–72 h	0	0.01
		8–16 h	25–72 h	0	0.02

No-AA—no acute appendicitis group, NAA—non-complicated acute appendicitis group, CAA—complicated acute appendicitis group, WBC—white blood cell, NEU—neutrophils, LYMP—lymphocytes, NEU/LYMP—neutrophil to lymphocyte ratio, CRP—C-reactive protein.

## Data Availability

Anonymized data are available from the senior author (TP) on reasonable request.
